# Current News and Opinion

**Published:** 1886-05

**Authors:** 


### ANNUAL COMMENCEMENT OF THE DENTAL DEPARTMENT OF THE UNIVERSITY OF MARYLAND.

The annual commencement of the University of Maryland, Dental Department, in connection with the seventy-ninth annual session of the University School of Medicine, was held at the Academy of Music, Baltimore, on Wednesday, March 17th, 1886, the reading of the mandamus and the announcement of the graduates by Prof. Ferdinand J. S. Gorgas, Dean. The degree of D. D. S. was conferred by Hon. S. Teackle Wallis, LL. D., Provost of the University, upon the following gentlemen, all of whom had attended two full sessions of five months each, in separate years:

Amend, Emil, Germany,
Baden, Frank A., Maryland,
Basehore, Horace E., Pennsylvania,
Brugeille, Emile, France,
Bookhart, Thomas W., South Carolina,
Bruce, William W., West Virginia,
Campbell, Oscar J., Virginia,
Chafee, Augustus H., South Carolina,
Diehl, John S., Pennsylvania,
Emerson, Joseph G., Brazil, S. Am.,
Furman, Charles Luff, New York,
Gasque, Elly A., South Carolina,
Greenawalt, A. H., D. D. S, Penn.,
Hartwig, Charles W., Maryland,
Hoffman, John H., Virginia,
Huggins, G. Allen, South Carolina,
Lowell, William H., Pennsylvania,
Lumsden, Frank H., Maryland,
Macgill, Lloyd T, Jr., Maryland,
Pleasants, Wilfred A., Virginia,
Proctor, Jr., W. Eppes, Virginia,
Purnell, Ralph C., Maryland,
Riley, James M, North Carolina,
Shields, Lewis N, Texas,
Sims, Benjamin F., South Carolina,
Slocum, Frank E., New York,
Wall, Joseph A, Pennsylvania.

The University Prize, gold medal, for the highest number of votes at the final examination, was awarded to William E. Proctor, Jr., of Virginia. Honorable mention was awarded to Wilfred A. Pleasants, of Virginia.

The address to the graduates was delivered by Col. William Allen, President of McDonough Institute.

The number of matriculants for the session of 1885–6 was ninety-one.

Although the number of matriculants for the session was larger than ever before, the number of graduates is smaller, on account of a strict compliance with the two-session rule, as a requisite for graduation, no less than twenty-two having been refused, who desired to graduate in one session, on five years or more of practice.

The annual meeting of the Alumni Association of the Dental Department, University of Maryland, was held at the Howard House, Thursday evening, March 18th Dr. Charles L. Steel, of Virginia, presided. Prof. F. J. S. Gorgas addressed the meeting. Members of the class of 1886 and a number of the graduates of other colleges were elected members. The following officers were elected for the ensuing year:

*President*—Dr. R. D. Dodson, Pennsylvania.
*Vice-President*—Dr. J. Fournien, New York.
*Secretary*—Dr. J. S. Kloeber, Virginia.
*Treasurer*—Dr. I. H. Davis, of Maryland.

### CHICAGO COLLEGE OF DENTAL SURGERY.

The fourth annual commencement exercises of the Chicago College of Dental Surgery were held at the First Methodist Church, Chicago, on Wednesday afternoon, March 31st, 1886.

The class valedictory was delivered by Robert E Moon, D. D. S., and the address to the graduates by W. L. Copeland, M. D., C. M., M. R. C. S., Professor of Anatomy.

The number of matriculates for the session was eighty-one, an increase of thirty-one over the previous course.

The degree of D. D. S. was conferred on the following graduates by Dr. James A. Swasey, President of the Board of Directors:

Harry Fenn Carson, Illinois.
Louis Chismann, Illinois.
Gilbert Walter Entsminger, Illinois.
Ernst August Huxmann, Illinois.
Joseph Perry Mertes, Wisconsin.
Robert Ellsworth Moon, Indiana.
James Stewart, Illinois.
Ellsworth Otis Whipple, New York.
Emory Melvil Cheadle, M. D., Oregon.
Joseph Grant Emery, Illinois.
Frank Eshbaugh, Illinois.
Henry Fredrick Marcoux, Illinois.
Theodore Felix Molt, Illinois.
Otto Henry Staehle, Illinois.
Thomas Benton Wheeler, Illinois.
Alfred Rogers Wilcox, Illinois.

The Secretary made his annual report as follows:

During the college year, which closes to-day, we have had eighty-one matriculates, which, compared with last year, shows an increase of thirty-one

Number of matriculates for the spring course, at the present time, forty-three.

Number of candidates for graduation, nineteen.

Number who passed a successful examination, sixteen.

Number of patients who have visited the infirmary for professional services, nine thousand and one.

These patients have had performed for them all of the usual dental operations, and many have been under treatment for surgical diseases of the mouth.

The number of instructors employed in the College is forty-two.

Of this number nine compose the regular faculty, eleven the Spring faculty, a superintendent of the infirmary, five demonstrators and sixteen clinical instructors.

During the last six months the number of lectures delivered was six hundred and twenty-four.

Number of clinical lectures, fifty-two.

Students entering this College must present evidence of having a good education in the English language.

An intermediate examination is required at the close of the junior year, for advanced standing and admission to the senior class.

The faculty having been frequently requested to advise young men wishing to enter the profession as to whether they should remain in a dentist's office a year before entering college, or begin at the college without previous training, have decided that some preliminary course is desirable, and to meet that want have organized the Spring course.

The Spring course is preliminary to the regular Winter course; it is, indeed, the beginning of the college year. It assumes that one entering the class is without any knowledge of dentistry, and yet it is prepared to meet the wants of students far advanced in their studies. It is intended to take the place of office instruction, and students without dental knowledge, as well as those advanced, who may wish to avail themselves of the great advantages offered for practical work and instruction, are earnestly advised to enter the Spring term. During the last week of each Spring term the student will be examined, and will receive certificates of attendance and grade of examination. This course cannot be considered as equivalent to a regular course of lectures in the requirements for graduation.

The growth of the College has been so rapid, and the increase in the number of students so great, that the Board of Directors have found it necessary to secure more commodious quarters. Accordingly, the building situated at the northeast corner of Madison street and Wabash avenue has been secured for a term of years. It has a frontage of sixty feet on Wabash avenue, and one hundred and sixty-five feet on Madison street, while the rear rests on Dearborn place, thus giving excellent light from three directions. It is supplied with passenger and freight elevators, and stairs in both front and rear.

The College will have a well-lighted and well-ventilated lecture room, faculty room and museum, a large room for the infirmary, with excellent light and having a capacity for sixty chairs, a large and well-fitted chemical laboratory, a mechanical laboratory and a complete physiological laboratory; also a dissecting room, patients' waiting room, students' cloak room and superintendent's room, together with closets, etc., etc.

When the work of fitting the building is finished the Chicago College of Dental Surgery will be in its appointments one of the most perfect and complete institutions of its kind extant. T. W. Brophy, Secretary.

---

### DENTAL JOURNALS WANTED.

Cash will be paid for the following numbers of dental journals, or an exchange will be made with those who desire to complete their own files:

The Dental Register:
Vols. III. and VI., complete.
Vol. XXXVI., No. 1.

American Journal of Dental Science (Third Series):
Vol. II., Nos. 6, 7, 8, 10.
" V., Nos. 3, 7, 11.
" VI., Nos. 2, 3, 5, 7, 10.
" VII., Nos. 2, 3, 7, 9.
" VIII., Nos. 6, 7, 10.
" XV., Nos. 3, 4.
" XVI., Nos. 1, 2, 4, 5, 6.

Missouri Dental Journal:
Vol. I., No. 3.
" V., Nos 5, 8, 9, 11. W. C. Barrett.

### ILLINOIS STATE DENTAL SOCIETY.

The twenty-second annual meeting will be held at Rock Island, commencing Tuesday, May 11th, 1886, and continuing four days.

REPORTS, ESSAYS AND DISCUSSIONS.

I. Report of Committee on Dental Science and Literature. Dr. Homer Judd, of Upper Alton, chairman.

II. Report of Committee on Dental Art and Inventions. Dr. J. Frank Marriner, of Ottawa, chairman.

III. Oral Surgery. Essay by Dr. John S. Marshall, of Chicago. Discussion opened by Dr. G. V. Black, of Jacksonville.

IV. Antiseptics and Disinfectants. Essay by Dr. A. W. Harlan, of Chicago. Discussion opened by Dr. Louis Ottofy, of Chicago.

V. The Retention of Pulpless Teeth in the Jaws. Essay by Dr. Homer Judd, of Upper Alton. Discussion opened by Dr. Geo. H Cushing, of Chicago,

VI. Preparation of Pulp-Canals and Cavities for Filling. Essay by Dr. C. R. Taylor, of Streator. Discussion opened by Dr J. A. W. Davis, of Galesburg.

VII. (Special Order for Thursday morning.) Report of Supervisor of Clinics, Dr. Frank H. Gardiner, of Chicago ; and Discussion on Operative Dentistry and General Practice. Opened by Dr. J. N. Crouse, of Chicago.

VIII. Post-Graduate Study. Essay by Dr. J. D. Moody, of Mendota. Discussion opened by Dr. Garrett Newkirk, of Chicago.

IX. Oral Chemistry. Essay by Dr. J. G. Reid, of Chicago. Discussion opened by Dr. W. A. Johnston, of Peoria.

---

### CHICAGO DENTAL SOCIETY.

At the annual meeting of the Chicago Dental Society, April 6th, the following officers were elected for the ensuing year:

*President*—Dr. Frank H. Gardiner.
*First Vice-President*—Dr. P. J. Kester.
*Second Vice-President*—Dr. W. B. Ames.
*Recording Secretary*—Dr. J. G. Reid.
*Corresponding Secretary*—Dr. A E. Matteson.
*Treasurer*—Dr. E. D. Swain.
*Librarian*—Dr. A. W. Harlan.
*Board of Censors*—Drs. L. L. Davis, J. W. Wassall, B. L. Rhein.
*Board of Directors*—Drs. Geo. H. Cushing, J. A. Swasey, E. Noyes.

A. E. Matteson, Corresponding Secretary.

---

### CONNECTICUT VALLEY DENTAL SOCIETY.

The semi-annual meting for 1886, of the Connecticut Valley Dental Society, will be held at Hartford, Conn., June 10th and 11th.

A cordial invitation is extended to all members of the profession to attend.

Geo. A. Maxfield, D. D. S.,
Secretary.

### FIRST DISTRICT DENTAL SOCIETY.

At the annual meeting, held April 7th, the following members were elected officers for the ensuing year:

*President*—Dr. William Carr.

*Vice-President*—Dr. J. F. P. Hodson.

*Secretary*—Dr. B. C. Nash.

*Treasurer*—Dr. Charles W. Miller.

*Librarian*—Dr. J. Bond Littig.

*Delegates to the Dental Society of the State of New York*—Drs. Alfred R. Starr and F. Austin Roy for four years; Dr. Martin C. Gottschaldt for three years, to fill vacancy.

---

### IMPOSTS ON DENTAL GOODS.

The foreign duties levied on imported dental materials are as follows:

| | Per cent. | | Per cent. |
|---|---|---|---|
| Artificial Teeth | 20 | Dental Rubber | 25 |
| Metallic Stoppings | 45 | Cement Stoppings | 20 |
| Gold or any Metal Stoppings | 45 | Gold for Plate, Wire, etc | 45 |
| Gold for Solders | 45 | Impression Wax | 20 |
| Artificial Teeth as Strings of Shades | 45 | Dental Instruments | 45 |
| Dental Tools | 45 | Mercury | 10 |
| Corundum | 20 | Dental Books | 25 |
| Burs and all Instruments used for the Dental Engine, including Engine | | | 45 |
| All Appliances containing Wood or Glass in their composition | | | 45 |

---

WHO WILL NOW SAY that Buffalo is not a medical center? Four Medical Journals are published here. We have two Medical Colleges, where twenty-two professors and twenty-one lecturers dispense medical lore. Besides the regular city and county societies, there are now four private medical clubs, not including the homeœpathic organizations.—*Buffalo Medical and Surgical Journal.*

Yes, and every alternate physician is connected with from one to four infirmaries, or dispensaries, or free hospitals, while the practitioner who has not secured an invitation to deliver a couse of free medical lectures before some literary, or social, or self-improvement society, is very lonesome indeed. If every man, woman and child in Buffalo is not thoroughly informed—or misinformed—concerning the mysteries of medicine, it surely is not the fault of the newly-fledged M. D's who are thirsting for distinction as lecturers.—*Ed.*

---

SCHOOL PHYSIOLOGY. A young lady's composition in one of the fashionable schools was as follows: Food is digested when we put it in our mouths. Our teeth chews it and our tongue rolls it down into our body. We should not eat so much bone-making food as flesh-forming and warmth-giving foods, for if we did we should have too many bones, and that would make us look funny.———

After all, this is but little more absurd than some of the queer things one occasionally hears in society meetings and—tell it not in Gath—sometimes sees in certain journals.—*Ed.*

No Thinking Person could confound the accomplished anatomical scholar, whose treatment of the mouth is based upon the principles of science, with the vulgar and greedy creature whose only object is to make a lucrative job for himself, and who is known willfully to damage the teeth of unhappy victims for the sake of extorting larger fees; but then the world is not made up of thinking people, and the quacks reap large harvests from the ignorance of the many. Fellows without other qualifications for their calling than a certain brutal strength and a certain empirical adroitness, and other fellows without even these advantages, and who declare that they neither expect nor desire to see their maltreated patients twice, manage by dint of puffing and impudence to gain large incomes at the expense of the fools who trust them. It is time that the real dentist should bestir himself.—*London Punch.*

---

Felix Weiss, L. D. S., of London, in the appendix to Vernon Galbray, tells the following anecdote:

"Very shortly after the writer of this book entered the profession, he had occasion to visit a celebrated dental depot for the purchase of materials. While there, a well-known man of the Vernon Galbray type entered and requested to be attended to. The principal of the establishment, however, refused to serve him. 'What do you mean,' the empire cried, greatly incensed. I simply mean that I refuse to serve you, for a man who can so lower himself by disreputable practices, and disgrace the profession as you are doing, shall never have it in his power to say that I assisted him.

"The gentleman who so willingly sacrificed his own personal interests to the general welfare of the profession was the late Claudius Ash."

---

Dr. Edward Berdoe, in the *British Medical Journal,* says that he has found carbolic acid and other antiseptics extremely useful in the treatment of that form of dyspepsia that is accompanied with acid eructations and gas, with pain after eating. The dose recommended is, five to ten minims of the glycerole of carbolic acid (one part crystals of carbolic acid to four parts glycerine) in mint water or other convenient vehicle ———

This form of dyspepsia is due to the stomach fungi of which Dr. Miller has given such a clear and interesting account in the last three numbers of the Independent Practitioner —*Ed*

---

Oh! Oh! That naughty *Weekly Medical Review* quotes from the March Independent Practitioner directions for obtaining a plaster cast of a nose, or other external organ, by painting the tissue over with melted paraffine, and thus wickedly says: "Be careful to prevent the paraffine from getting into the eyes of the victim, or the use of a 'paraffine' optics will be impaired."

---

Wolves are more often subject to hydrophobia than even dogs, and in Russia many persons are annually bitten by mad wolves. A number of Russian Mujiks, who had been bitten by a mad wolf, were recently subjected to treatment by M. Pasteur. One of them died, and in his malar bone was found a large splinter from *a decayed tooth* of the wolf that had bitten him.

Billroth, the eminent pathologist, writes the following on antiseptics:

1. Iodoform is the safest and most effective of all manageable antiseptics.
2. Moss, wood-turf, mould, and oakum are useful when there are discharges from the wound.
3. Corrosive sublimate in dilute solution is practically inert as an antiseptic to wounds, and renders the patient and surgeon alike liable to mercurial poisoning.
4. Carbolic acid, which is known to be dangerous in strong solutions, in very weak ones is as good for wound irrigation as clean water, but probably no better.

---

The first charge to a jury of a newly elected justice was as follows, "Gentlemen of the jury, this is new business for me, as this is my first case. You have heard all the evidence; you have also heard what the learned counsel have said. If you believe what the counsel for the plaintiff has told you, your verdict will be for the plaintiff; but if, on the other hand, you believe what the defendant's counsel has said, then your verdict will be for the defendant; but if you are like me, and don't believe a word that either of them has said, then I'll be hanged if I know what you will do. Constable, take charge of the jury."

---

M. Laffont has studied the influence of nitrous oxide (anæsthesia being deep and prolonged) on respiration and the functions of the heart and liver. Sugar appears in the urine, and phenomena of asphyxia are observed. Respiratory movements increase in frequency and amplitude under the first inhalations, and at the moment of the anæsthesia the breathing is panting. The heart-beats are increased at the beginning, but soon become slow. M. Laffont therefore concludes that the state of anæsthesia induced by this gas is by no means harmless. —*Progres Medical.*

---

Dr. Leon F. Harvey, of Buffalo, has retired from practice, his health being too precarious to permit of the cares of an active business. Dr. Harvey has long occupied a prominent place in dentistry, and it is a source of regret that he is lost to the profession. The elder Harvey—Dr. C. W.—was one of the pioneers of dentistry in Buffalo. Like his son, he attained to eminence in his profession, and still lives to enjoy the competency which was the reward of his skill and industry.

---

The Independent Practitioner, with the June number, will come out in an entire new dress. The type in which the body of the journal has been set has become somewhat worn, and it will be cast aside and a new font employed. The publishers have never spared labor or expense when any means for improvement were suggested, and they believe that in its new dress this journal will as much excel in beauty as it has in excellence.

---

The St. Louis Medical and Surgical Journal says that a gentleman who has little faith in anatomical study writes it an account of a wonderful case of intestinal obstruction, which he finally cured by passing a piece of rubber-tubing up into the rectum just as long as he could shove it in. He says he "passed nearly two yards of tubing into the receptacle," and demands "What I want to know is, *is* there such a thing as the illiasecle valve?"

DR. E. H. GRIFFIN, of New York City, reports the case of a patient, aged 32, who had a tooth, the crown of which projected into the nose. There was a congenital cleft of both the hard and soft palates. The root of the lateral incisor on one side was found in the gum tissue, while the crown, in full development, was in the nasal cavity. The patient said the tooth had been there for years, but had never given him any trouble, and he declined to have it removed.

---

THE FOLLOWING BILL has been introduced in the New York Legislature:

SECTION 1. A dentist who shall administer chloroform or ether to any person unless said dentist shall be a regularly graduated physician from some legally incorporated school of medicine and surgery, is guilty of a misdemeanor.

SEC. 2. This act shall take effect on and after the first day of September, eighteen hundred and eighty-six.

---

DR. A. W. HARLAN, of Chicago, is translating the later work of Magitot, which forms the concluding portion of Legros and Magitot's "The Dental Follicle," translated by the lamented Dean. Dr. Harlan is well qualified for the work, as well from his personal qualifications as from his long association with Dr. Dean.

---

DR. B. H. TEAGUE, of Aiken, South Carolina, has devised a new engine disk, made of paper and cloth, with a depressed centre, which greatly facilitates the dressing of fillings or teeth near the cervical border. They are covered with pumice or emery, and are a decided improvement over the old flat disk.

---

THE EFFECTS OF THE SOUTHWESTERN RAILROAD STRIKE are widespread. The *Iowa State Medical Reporter* for January arrived April 12th. The explanation given by the publishers is that their paper, of an unusual tint, is manufactured in St. Louis, and the strike interfered with its delivery.

---

THE LANCET AND CLINIC says there is a man named Smith living in one of the small towns in Michigan, who claims to be a dentist. A sign over his door, painted by himself, reads: "Teeth Extracted Without Enny Pane Laffin Gas Ten (10) cents a Ha Ha."

---

DR. D. G. BRINTON, the accomplished editor of *The Medical and Surgical Reporter*, has been chosen laureate of The Sociëté Americaine de France, and has been awarded the medal of the society for his work on the aboriginal tongues of America.

---

DR J. SMITH DODGE, JR., uses instruments made of platinum wire for carrying medicated cotton into root canals. They are so pliable that they readily conform to the openings in the roots, and they neither oxidize nor blacken.

---

DR FRANCIS finds Donaldson's scrapers for removing debris from pulp canals exceedingly useful. They also do good service in removing bits of cotton that have been packed into roots.

---

AN EXCELLENT CEMENT for mending broken plaster casts may be made by dissolving celluloid in a saturated solution of camphor-gum in alcohol, until a paste is obtained.

